# The Race to Save Lives: Demonstrating the Use of Social Media for Search and Rescue Operations

**DOI:** 10.1371/currents.dis.806848c38f18c6b7b0037fae3cd4edc5

**Published:** 2014-11-06

**Authors:** Tomer Simon, Bruria Adini, Mohammed El-Hadid, Avishay Goldberg, Limor Aharonson-Daniel

**Affiliations:** Recanati School for Community Health Professions, Faculty of Health Sciences, Ben-Gurion University of the Negev; Ready.org.il – Emergency readiness and preparedness in Israel; PREPARED Center for Emergency Response Research, Ben-Gurion University of the Negev, Beer Sheba, Israel; Jordan Red Crescent, Amman, Jordan; Ben-Gurion University of the Negev, Beer Sheva, Israel; Ben-Gurion University of the Negev, Beer Sheva, Israel

## Abstract

Importance: Utilizing social media in an emergency can enhance abilities to locate and evacuate casualties more rapidly and effectively, and can contribute towards saving lives following a disaster, through better coordination and collaboration between search and rescue teams. Objective: An exercise was conducted in order to test a standard operating procedure (SOP) designed to leverage social media use in response to an earthquake, and study whether social media can improve joint Israeli-Jordanian search and rescue operations following a regional earthquake. Design: First responders from both Jordan and Israel were divided into two mixed groups of eight people each, representing joint (Israeli-Jordanian) EMS teams. Simulated patients were dispersed throughout the Ben-Gurion University Campus. The first search and rescue team used conventional methods, while the second team also used social media channels (Facebook and Twitter) to leverage search and rescue operations. Participants: Eighteen EMS and medical professionals from Israel and Jordan, which are members of the Emergency Response Development and Strategy Forum working group, participated in the exercise. Results: The social media team found significantly more mock casualties, 21 out of 22 (95.45%) while the no-media team found only 19 out of 22 (86.36%). Fourteen patients (63.63%) were found by the social media team earlier than the no-media team. The differences between the two groups were analyzed using the Mann-Whitney U-test, and evacuation proved to be significantly quicker in the group that had access to social media. The differences between the three injury severities groups' extraction times in each group were analyzed using the Kruskal-Wallis test for variance. Injury severity influenced the evacuation times in the social media team but no such difference was noted in the no-media team. Conclusions: Utilizing social media in an emergency situation enables to locate and evacuate casualties more rapidly and effectively. Social media can contribute towards saving lives during a disaster, in national and bi-national circumstances. Due to the small numbers in the groups, this finding requires further verification on a larger study cohort.

## Background

Earthquake casualties' are often characterized by multisystem injuries, including limb fractures, crush syndrome, burns and injuries to major and vital organs (i.e. head and spine) 1 ^,^ 1 ^,^ 2 ^,^ 3 ^,^ 4. As initially suggested by Shultz et al in 1996 5, two recent studies have proved once more that immediate medical response to earthquake victims can improve their outcome substantially 6 ^,^ 7. Research groups worldwide strive to improve urban search and rescue efforts following a disaster. In the past decade, these efforts have been increasingly supported by information systems and technologies 8 ^,^ 9 ^,^ 10. Geo-referencing information that is easily accessible can reduce the search areas considerably in rescue operations 11. Social media can provide two types of location based information: geo-location data which is clearly identifiable information; and location-referencing that uses one place as a replacement for another or mention of location via a landmark 12 ^,^ 13. Rapid assessment of critical information, such as the area affected, and specific locations where search and rescue missions are likely to be required, are of high priority in emergency management 14. During and immediately following a disaster, conventional means of communication often become unavailable, and alternative mechanisms such as social media networks become an important channel for information gathering and sharing 15 ^,^ 16. Effective bilateral sharing of information becomes even more crucial in cross border collaboration following a disaster. The aim of the current study was to examine the potential contribution of social media, to search and rescue teams, particularly in cross border collaboration in response to an earthquake.

## Methods

In November 2012 the joint Jordanian-Israeli Development Strategic Forum (DSF) convened to conclude their tri-annual activities to enhance emergency preparedness. The DSF is comprised of emergency management experts from Jordan and Israel, consisting of field practitioners from emergency medical services (EMS), senior policy makers as well as academic researchers. In order to study the contribution of social media for managing a potential earthquake in the region, a joint exercise was developed and implemented. The exercise provided an opportunity for Jordanian and Israeli EMS personnel to collaborate in order to respond to a simulated disaster. First responders from both Jordan and Israel were divided into two mixed groups of eight people each, representing joint (Israeli-Jordanian) EMS teams. The exercise's objective was to verify and test a standard operating procedure (SOP) designed to leverage social media in response to an earthquake. The exercise was conducted at the campus of the Joyce and Irving Goldman Medical School and Health Sciences in Ben-Gurion University of the Negev in Beer-Sheba (BGU). Figure 1 shows the map of BGU’s main campus, where the black dashed rectangle indicates the location of the Health Sciences campus where the exercise was held. Simulated patients were dispersed throughout the campus and its six buildings, coordinated through the faculty administration office. They were placed in class rooms, staff offices, corridors and in open spaces.

**The Ben-Gurion University Campus:**
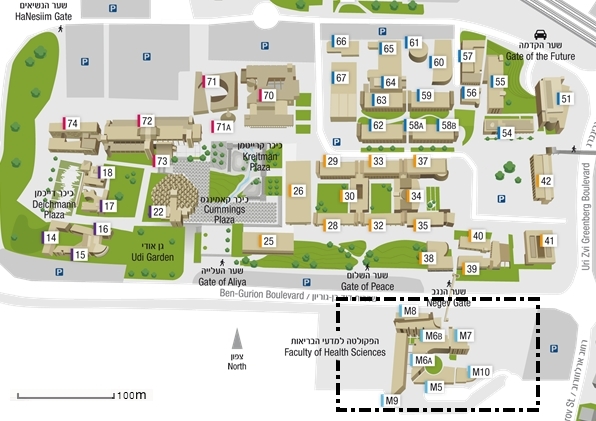
The black dashed line represent the area where the exercise took place

Both teams were sent out to "search and rescue" mock casualties throughout the campus. The first team ("No Media Team" - NMT) used only conventional methods of search and rescue, mainly spreading staff simultaneously to different locations to verify reconnaissance of all casualties, communicating through radio and cellular channels, as well as with the Zello (http://zello.com/) push-to-talk (PTT) application, which provided a walkie-talkie experience. The second team ("Social Media Team" - SMT) used both conventional methods as well as social media channels (Facebook and Twitter) to leverage their search and rescue operations. Both teams were briefed regarding the exercise and its goals.

Figure 2 describe the involved players, the operational model, and the information flow of the exercise. The solid lines represent direct command and control actions, while the dashed lines signify information flow (publish, receive and search for updates) and communication channels. The exercise included five types of participants: 1) Players- personnel who have an active role in responding to the simulated emergency and perform their regular roles and responsibilities during the exercise. 2) Controllers- who set up and operate the exercise site, plan and manage its execution, and simulate roles of response individuals and agencies not represented in the exercise. 3) Simulators- control staff personnel who operate out of the Simulation Cell, enacting roles in accordance with instructions and information provided in the Master Scenario Events List (MSEL). 4) Evaluators- who assess and document participants’ performance based on established emergency plans and exercise evaluation criteria. 5) Simulants- volunteers who simulate specific roles during the exercise, mainly the mock casualties.

**Exercise information flow and operational model.**
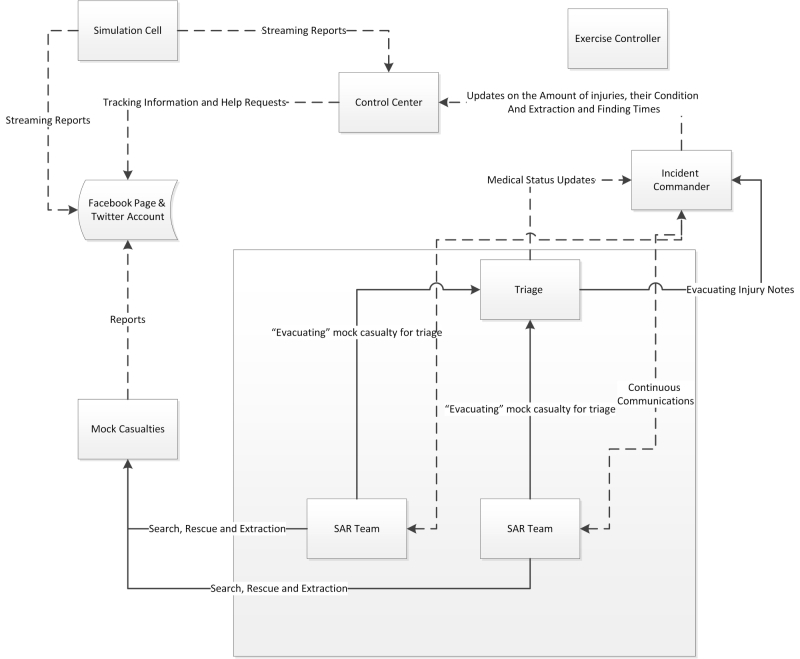
Solid lines represent command and control and the dashed line represents the information flow and communications

Both the NMT and SMT operated under the same hierarchical structure, which included a control center, incident commander, triage center and two SAR teams. The control center managed the work and progress of the entire team’s operation during the exercise. Each control center was allocated an office in which they deployed an operation center. An incident commander was appointed by each team whose responsibility was to coordinate search and rescue operations of the SAR teams from the field.

A Facebook Group was created for the purpose of the exercise, but only the participants of the social media team were approved to join it. The Simulation Cell, which was in charge for role-playing nonparticipating organizations or individuals and for information dissemination to the exercise participants used a Twitter account that provided a conduit through which they could publish information. The exercise duration was planned for 90 minutes, or until one of the teams has found all of the mock casualties. All information and directions to the participants were communicated from the Simulation Cell, according to a predefined MSEL, which included a scripted chronological sequence of events and actions to be injected into the exercise by the simulation cell. The command center of both teams updated in real time a spreadsheet, through Google Docs [1]. This spreadsheet was visible to the exercise directors sitting in the exercise headquarters. The SMT were encouraged to use social media for both receiving and updating information during the exercise. Active interaction with the Simulation Cell was rewarded as it provided more operational information. Twenty two students representing mock casualties, were assigned with injury tags describing their medical condition, and were distributed to 20 different locations in a radius of approximately two square kilometers within the campus. Both exercise teams were instructed to locate and extract the casualties, implement triage processes, state the injury severity (severe/moderate/mild) and "evacuate" the mock casualties to a hospital according to priorities (evacuation in the exercise meant bringing the injury tag of the mock casualty to the command center). Furthermore, they were instructed to record the location of each simulated casualty, time of extraction and evacuation, and the ID of each simulated patient. Only mock casualties with smartphones were allowed to publish information (visual and/or textual) on Facebook. This information could include a general description of their location (i.e. second floor of the X building), without specifying their exact position. Also, they could upload photos of their location using their smartphones. The simulation cell could publish additional information concerning the casualties' locations, but only items that would in such situations be published by bystanders. The SMT team was encouraged to read the tweets and Facebook posts and coordinate their search and rescue efforts accordingly.

Minimum, maximum and mean times (duration) in minutes from exercise commencement to the extraction were calculated and compared between groups. In addition, the total number of casualties found and rescued by each group was compared. Statistical Package for the Social Sciences (SPSS) 18 was used for the analysis. As the number of records was small (22 maximum) and normalcy could not be assumed, a non-parametric (Mann-Whitney) test was used to compare between the groups. An additional comparison was conducted between the three levels of injury severity within the casualties' array of each team. A non-parametric test (Kruskal-Wallis) was used to compare the three injury severity groups' extraction times between media groups.

## Results

During the exercise, the Simulation Cell published location based information, through both the Facebook group and the Twitter account, regarding the possible location of the simulated casualties. Six of the eight SMT members had a smartphone with which they could check the above mentioned social media channels for information throughout the exercise. Two team members mentioned that they had difficulties using both Facebook and Twitter concurrently on their smartphone, as it required them to switch between the applications repeatedly. Up to the end of the exercise, the SMT found 21 out of 22 (95.45%) simulated patients, while the NMT found only 19 out of 22 (86.36%). Furthermore, the SMT found the first patient five minutes earlier than the NMT, and the last patient seven minutes earlier than the NMT: ten minutes versus 15 minutes for first patient and 55 minutes versus 62 minutes for last patient. Time statistics are presented in table 1.

**Table 1: Distribution of extraction times:** 

**Media Availability Group**	**Number (%) of casualties found (N= 22)**	**Range (time span) for patient extraction (minutes)**	**Mean Time** **(minutes)**	**Standard deviation (minutes)**
SMT	21 (95.5%)	10-55	27.2	12.2
NMT	19 (86.4%)	15-62	40.0	15.5

Figure 1 summarizes and compares both teams' patient location and extraction times.

The dotted line represents the median time of the NMT, until which the SMT located and rescued 17 simulated patients (77.27%) while the NMT located 8 patients (36.36%). Fourteen patients (63.63%) were found by the SMT earlier than the NMT (including two that the NMT did not locate at all). Only four patients (18.18%) were located by the NMT prior to their location by the SMT. The gap between the solid (SMT) and the dotted (NMT) lines demonstrates the difference between the median extraction times of each group.

**Time of extraction of mock casualties.**
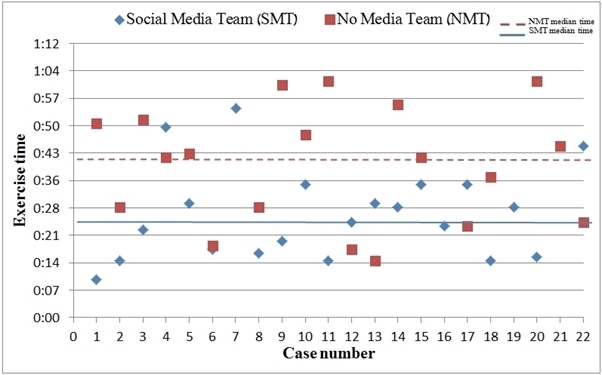
The graph presents the casualty's number (on the X axis) and the time taken to locate them in minutes (on the Y axis)

The differences between the two groups were analyzed using the Mann-Whitney U-test; the null hypothesis being that there would be no difference in the distribution of times to evacuation between the two groups. This hypothesis was rejected as statistically significant differences were identified (p=0.011). Evacuation proved to be significantly quicker in the group that had access to social media.

Each team nominated a medical professional who was responsible for the simulated patient's medical triage following extraction. Patient severity was assigned as one of three categories: Severe, Moderate, and Mild.

Before comparing the times to evacuation by severity category, we compared severity assignment between media availability groups in order to verify that there were no significant differences between the groups in regard to mock patient severity. A chi-square test confirmed that no significant differences existed in severity between the SMT and NMT in regard to the assignment of severity categories.

The differences between the three injury severities groups' extraction times in each social media group were then analyzed using the Kruskal-Wallis test for variance.

The null hypothesis was that the distribution of the time would be the same across the categories of severity. This hypothesis was rejected in the SMT (p=0.029) and retained in the NMT (p=0.762) meaning that injury severity influenced the evacuation times in the SMT team but no such difference was observed in the NMT team.

## Discussion

Recurrence of preventable dysfunction during disasters, as was displayed in Hurricane Sandy (2012), Hurricane Katrina (2005) and Tropical Storm Allison (2001), displays the vital need for raising awareness and increase emergency preparedness 17.

Rescue time is a crucial lifesaving component in responding to earthquakes 18 ^,^ 19; therefore, seeking new ways to improve the ability to search and rescue, such as through the utilization of social media, is of crucial importance. Raising awareness and practicing the skills necessary for improved rescue abilities can be achieved via exercises such as the one implemented in this study 20 .

Exercises that leverage and integrate social media expose the practitioners to these mechanisms, emphasizing their challenges and opportunities. Social media should be integrated into disaster management activities 21. As this is a developing and novel field, the combination and utilization of social media as part of the emergency response activities has been scarcely exercised before. The current exercise is innovative in two important aspects; the first being that the exercise was performed by actual emergency responders, performing search and rescue operations to locate and extract simulated trapped casualties. The second is the collaboration between two foreign emergency organizations, cooperating in a mixed response teams' mode.

The exercise enabled to compare performance of two first responders' teams, engaging in identical tasks, utilizing similar manpower and logistic resources, differing solely in the availability of social media capacities. Thus, the advantages achieved through the use of the social media in the search and rescue activities could be well displayed and understood. While most of the former studies focused on the integration of one specific type of social media and its influence on staff performance, the present study used both Facebook and Twitter[1] as channels for information dissemination. Abbasi, et al. 21 tried to evaluate different collection and filtering software and techniques of the tweets. Conversely, the current exercise incorporated social media as an integral tool for the emergency responders that used and accessed the information using their smartphones while performing search and rescue operations throughout the campus.

The significant gaps in performance between the two groups (with and without social media) displayed that social media may and should have a major impact on the search and rescue operations as means of intra and inter-team collaboration, sharing information and optimizing resources utilization. This media can facilitate not only local and national response, but also a cross-border regional response, enabling to overcome obstacles such as language barriers or different modes of operation 20.

Communication is one of the fundamental tools of emergency management. Communication systems must be able to withstand a disaster and enable devices to function effectively even when communication networks have collapsed 22. Most disasters cause severe damage to communication infrastructure 23. Phone switches and cell phone towers might collapse, fully or partially, thus disrupting the much needed communication 24. Severe natural disasters may cause the entire communications grid to blackout, as infrastructure is severely damaged 25. As the conventional means of communication become irrelevant during and immediately following a disaster, alternate means such as social networks become an important conduit for information gathering and sharing 26 ^,^ 27 ^,^ 28. Research shows that even though communication networks collapse, the internet continues to operate and to enable communication channels even when other channels are disrupted due to the disaster. This phenomenon was observed during the Chilean earthquake in 2010 29, and in following the 2011 earthquake and tsunami in Japan 30. Nevertheless, in events in which the communication infrastructure collapses, emergency responders will need to utilize traditional radio-based communication technology, as well as runners to convey information and updates 31.

## Limitations

Although this study and exercise were carefully planned and executed, two limitations should be noted. The first one is the limited, relatively small number of mock casualties that prevented use of statistical tests that are based on normal distribution. Second, although various severe disasters that occurred worldwide in recent years have demonstrated the internet's survivability, communication and power infrastructures may collapse, thus making social media unavailable.

## Conclusions

Social media provides a standardized and familiar platform that can serve to create bridges, offering options for cooperation, coordination and collaboration in order to improve preparedness and response to emergencies. Utilizing social media in an emergency, as was well displayed during the exercise conducted in the present study, enabled to locate and evacuate casualties more rapidly and effectively. Thus, social media can contribute towards saving lives during a disaster. Nevertheless, it is recommended that the social media emergency exercise be further implemented and validated in other emergency scenarios.

## Competing Interests

The authors have declared that no competing interests exist.
